# The Human Nose Organoid Respiratory Virus Model: an *Ex Vivo* Human Challenge Model To Study Respiratory Syncytial Virus (RSV) and Severe Acute Respiratory Syndrome Coronavirus 2 (SARS-CoV-2) Pathogenesis and Evaluate Therapeutics

**DOI:** 10.1128/mbio.03511-21

**Published:** 2022-02-15

**Authors:** Anubama Rajan, Ashley Morgan Weaver, Gina Marie Aloisio, Joseph Jelinski, Hannah L. Johnson, Susan F. Venable, Trevor McBride, Letisha Aideyan, Felipe-Andrés Piedra, Xunyan Ye, Ernestina Melicoff-Portillo, Malli Rama Kanthi Yerramilli, Xi-Lei Zeng, Michael A. Mancini, Fabio Stossi, Anthony W. Maresso, Shalaka A. Kotkar, Mary K. Estes, Sarah Blutt, Vasanthi Avadhanula, Pedro A. Piedra

**Affiliations:** a Department of Molecular Virology and Microbiology, Baylor College of Medicine, Houston, Texas, USA; b Advanced Technology Cores, Baylor College of Medicine, Houston, Texas, USA; c Department of Molecular and Cellular Biology, Baylor College of Medicine, Houston, Texas, USA; d Department of Pathology, Baylor College of Medicine, Houston, Texas, USA; e Department of Pediatrics, Pulmonary Medicine Service, Baylor College of Medicine, Houston, Texas, USA; f Environmental Safety Department, Baylor College of Medicine, Houston, Texas, USA; g Department of Pediatrics, Baylor College of Medicine, Houston, Texas, USA; h Department of Medicine, Section of Infectious Diseases and Gastroenterology, Baylor College of Medicine, Houston, Texas, USA; i Department of Medicine, Section of Gastroenterology and Hepatology, Baylor College of Medicine, Houston, Texas, USA; Dartmouth College

**Keywords:** nose organoids, respiratory syncytial virus (RSV), severe acute respiratory syndrome coronavirus 2 (SARS-CoV-2), cilia, palivizumab, air-liquid interface (ALI) culture, epithelium, airway, mucus, therapeutics, cytokines, ALI cultures, airway organoids, immunoprophylaxis, RSV, SARS-CoV-2

## Abstract

There is an unmet need for preclinical models to understand the pathogenesis of human respiratory viruses and predict responsiveness to immunotherapies. Airway organoids can serve as an *ex vivo* human airway model to study respiratory viral pathogenesis; however, they rely on invasive techniques to obtain patient samples. Here, we report a noninvasive technique to generate human nose organoids (HNOs) as an alternative to biopsy-derived organoids. We made air-liquid interface (ALI) cultures from HNOs and assessed infection with two major human respiratory viruses, respiratory syncytial virus (RSV) and severe acute respiratory syndrome coronavirus 2 (SARS-CoV-2). Infected HNO-ALI cultures recapitulate aspects of RSV and SARS-CoV-2 infection, including viral shedding, ciliary damage, innate immune responses, and mucus hypersecretion. Next, we evaluated the feasibility of the HNO-ALI respiratory virus model system to test the efficacy of palivizumab to prevent RSV infection. Palivizumab was administered in the basolateral compartment (circulation), while viral infection occurred in the apical ciliated cells (airways), simulating the events in infants. In our model, palivizumab effectively prevented RSV infection in a concentration-dependent manner. Thus, the HNO-ALI model can serve as an alternative to lung organoids to study respiratory viruses and test therapeutics.

## INTRODUCTION

With the onset of the coronavirus disease 2019 (COVID-19) pandemic, it is imperative now more than ever to have preclinical, physiologically relevant airway models to evaluate viral infection and potential therapeutics. Airway organoid (AO) models have quickly become an important investigational tool due to their ability to mimic human respiratory physiology (1). Previous studies have reported culturing AOs from human-induced pluripotent stem (iPS) cells that represent the fetal or developmental stages of the lung (2–6), and the Clevers group recently reported an advanced method for long-term culturing of human lung tissue-derived AOs (7). Brewington et al. (8) and Gamage et al. (9) also modeled nasal epithelial cells using nasal biopsy samples and nasal brushings. However, all the above-mentioned methods utilize invasive techniques and typically require physicians to obtain lung tissue or bronchoalveolar lavage fluid or nasal brushings from patients, which limit their application to the general researchers and for therapeutic screening. Therefore, a critical need remains for the development of a noninvasive method for generating AOs that can be readily applied to both pediatric and adult populations. Here, we report a novel, expandable, *ex vivo* human nose organoid (HNO) model that capitalizes on noninvasive techniques and yet retains the architecture of the respiratory epithelium. Additionally, we have effectively modeled these nasal wash- and swab-derived HNOs to study the pathogenies of the major pediatric respiratory viral pathogen, respiratory syncytial virus (RSV), and the foremost global respiratory viral pathogen, severe acute respiratory syndrome coronavirus-2 (SARS-CoV-2).

Globally, RSV infection in children <5 years old results in 33.1 million cases, 3.2 million hospitalizations, and up to 200,000 deaths, annually (10, 11). RSV is the major cause of acute lower respiratory tract illness (ALRTI) in children, accounting for approximately 20% of all ALRTI (12). RSV infects almost all children by 2 years of age and causes repeated reinfections throughout life (13). RSV is also a significant cause of respiratory disease morbidity and mortality in older adults, immunocompromised adults, and those with chronic pulmonary disease (14). Unlike RSV, the SARS-CoV-2 pandemic has resulted in a record-breaking global disaster. The rapidly spreading SARS-CoV-2 has caused over 256 million cases and 5.1 million deaths as of November 2021 (15). Therefore, it is important to develop human model systems to study viral pathology and to test therapeutics against them. In this study, we modeled an HNO-derived air-liquid interface (ALI) culture system to grow and study RSV and SARS-CoV-2 infections. The HNO-ALI cultures were readily infected by contemporaneous RSV strains (RSV/A/Ontario [ON] and RSV/B/Buenos Aires [BA]) and SARS-CoV-2 (WA-1), resulting in epithelial damage reminiscent of human pathology. Cytokine analysis of RSV- and SARS-CoV-2-infected HNO-ALI epithelia demonstrated a cell polarity-specific response, thus highlighting the importance of using polarized cells to understand the host immune response. Furthermore, this HNO-ALI respiratory virus model functions as an *ex vivo* human challenge model where we tested the efficacy of palivizumab, a monoclonal antibody used for the prevention of RSV.

## RESULTS

### Development of the human nose organoids (HNOs).

**FIG 1:**
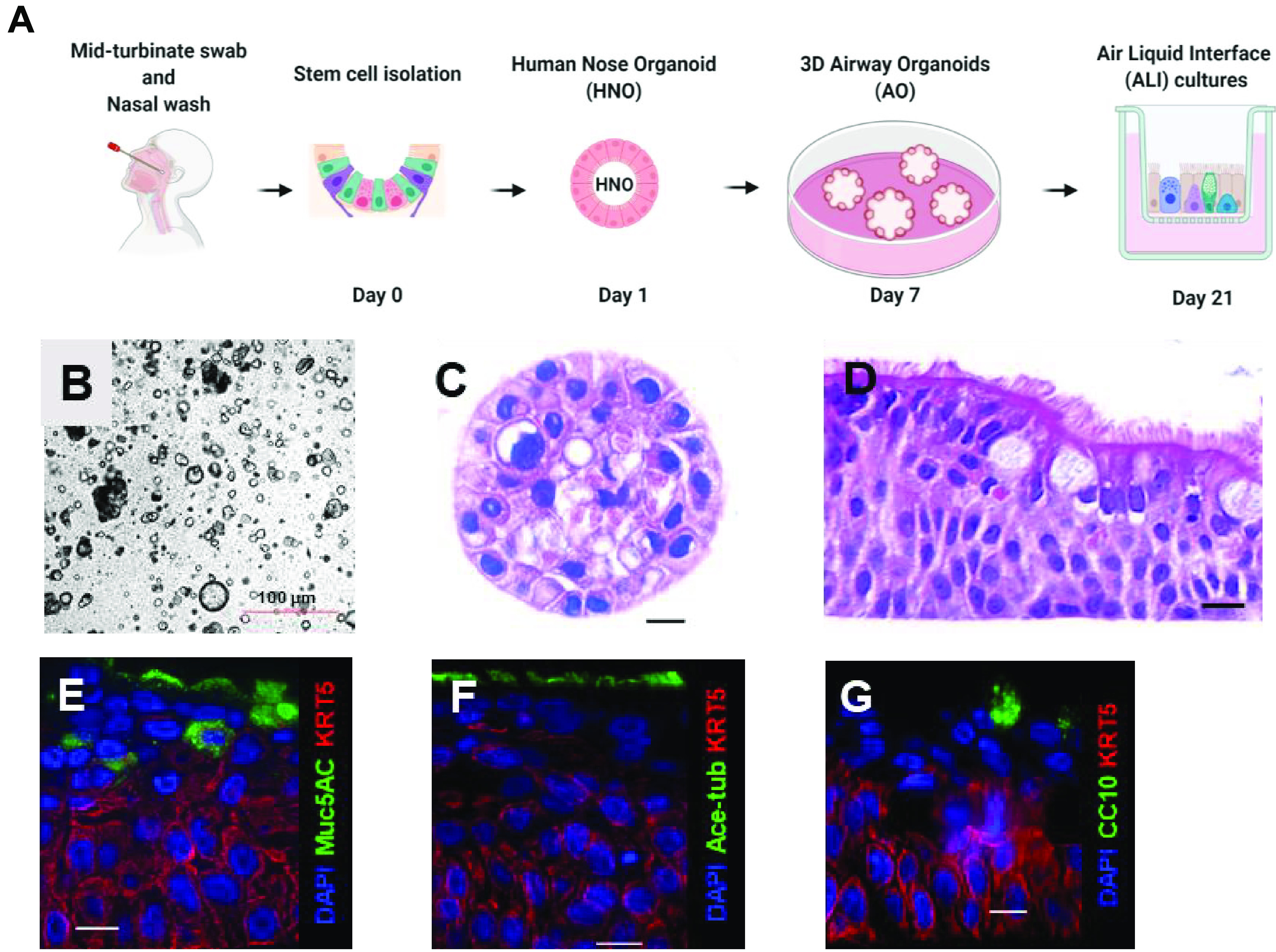
Derivation and characterization of human nose organoids (HNOs). (A) Schematic representation of the workflow for making of HNOs. (B) Bright-field image of 3D HNOs in culture. Scale bar equals 100 μm. (C) Hematoxylin and eosin (H&E) staining of 3D HNOs. (D) H&E staining of pseudostratified airway epithelium of HNO-ALI culture. (E to G). Immunofluorescence of HNOs. Basal cells are labeled by keratin 5 (KRT5), in red. DAPI stains each nucleus. (E) Goblet cells are shown in green, labeled by mucin 5AC (MUC5AC). (F) Ciliated epithelium is shown in green, labeled by acetylated alpha tubulin (Ace-tubulin) and (G) club cells in green, labeled by CC10. Scale bar equals 10 μm.

We established five lines of HNOs using stem cells isolated from nasal wash and midturbinate swab samples collected from human volunteers adopted from a recently published protocol (7) (Fig. 1A). The three-dimensional (3D) HNOs formed between 2 and 3 weeks after initial adaptation of stem cells to specialized growth medium conditions (Fig. 1B and C). Microscopically, the differentiated HNO-ALI culture system was composed of polarized, pseudostratified airway epithelia containing basal cells (keratin 5 positive, KRT5), secretory club cells SCGB1A1 (CC10 positive), goblet cells (mucin 5AC positive, Muc-5AC), and ciliated cells (acetylated tubulin positive, Ace-tub) (Fig. 1D to G). Beating cilia of HNO-ALI were also visible under light microscopy. We performed RNA sequencing of undifferentiated 3D HNOS and early (21-day) and late differentiated (31-day) HNO-ALI to analyze airway cell-specific gene expression patterns. Our data reveal that the HNO-ALI transcriptome was highly enriched for several of the airway epithelial cell-specific markers, including keratins, dynein, and secretoglobins (Data Set S1 and S2) and also showed hallmarks of ciliary function as shown by gene set enrichment analysis (GSEA) (Fig. S1). In summary, our HNOs retained the *in vivo* characteristics of human airway epithelia and can also be indefinitely passaged and frozen for long-term expansion.

10.1128/mbio.03511-21.1 FIG S1 List of ciliary epithelia associated genes in human nose organoids (HNOs). The heatmap summarizes a list of expression of ciliary-associated genes in undifferentiated 3D AO cultures and 21-day differentiated ALI cultures. This is suggestive of an effective differentiation process inducing ciliogenesis and functional ciliary movements in HNO-ALI cultures. Download FIG S1, PDF file, 0.01 MB. Copyright © 2022 Rajan et al. 2022 Rajan et al. https://creativecommons.org/licenses/by/4.0/ This content is distributed under the terms of the Creative Commons Attribution 4.0 International license.

10.1128/mbio.03511-21.7 DATA SET S1 RNA-Seq gene counts of HNOs. Download Data Set S1, XLSX file, 5.3 MB. Copyright © 2022 Rajan et al. 2022 Rajan et al. https://creativecommons.org/licenses/by/4.0/ This content is distributed under the terms of the Creative Commons Attribution 4.0 International license.

10.1128/mbio.03511-21.8 DATA SET S2 GSEA summary of differentially regulated genes of 3D HNOs and HNO-ALI. Download Data Set S2, XLSX file, 2.7 MB. Copyright © 2022 Rajan et al. 2022 Rajan et al. https://creativecommons.org/licenses/by/4.0/ This content is distributed under the terms of the Creative Commons Attribution 4.0 International license.

### Replication kinetics of RSV and SARS-CoV-2 in the HNO-ALI system.

**FIG 2:**
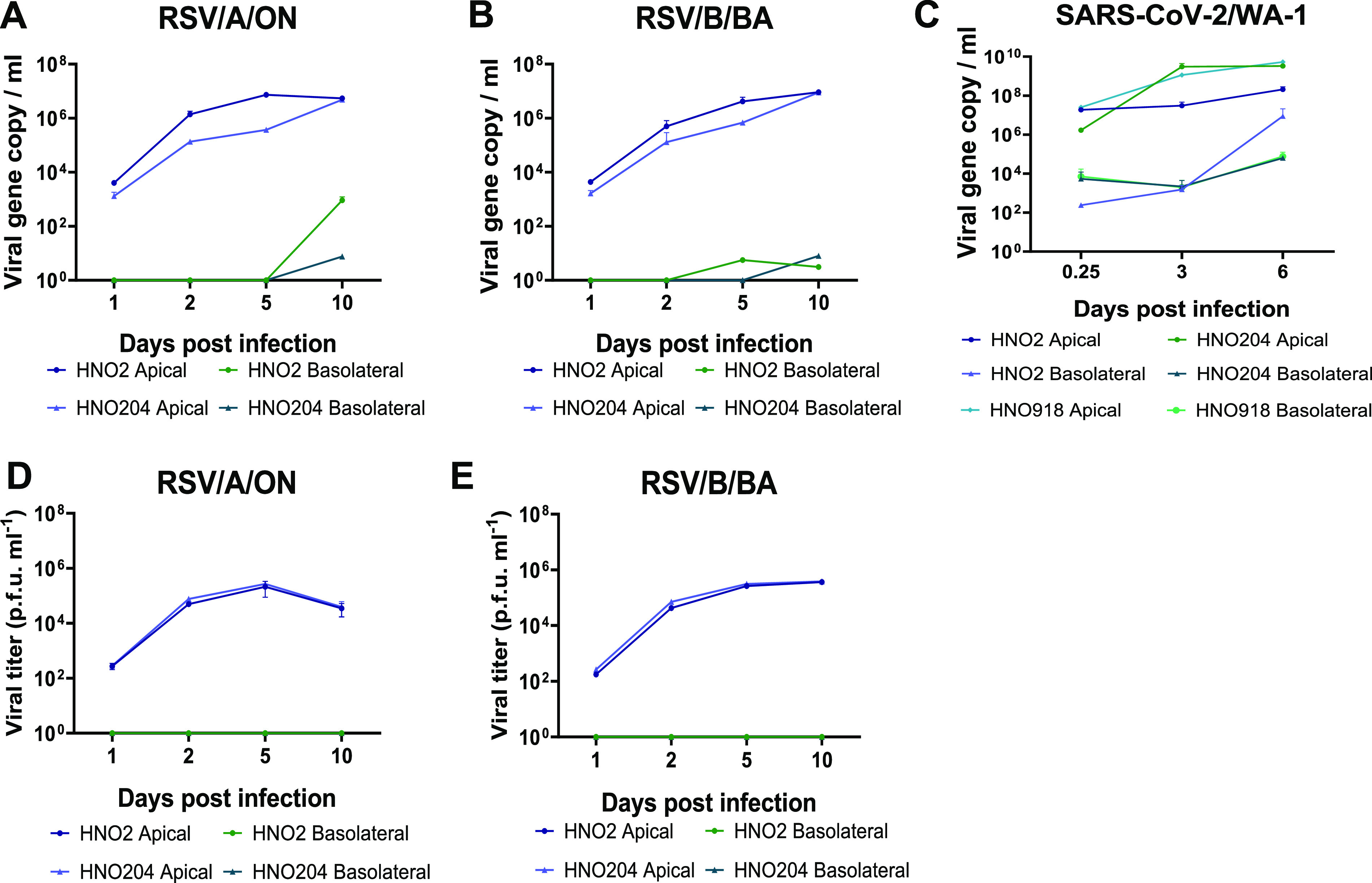
Infection of human nose organoids (HNOs) with respiratory syncytial virus (RSV) and severe acute respiratory syndrome coronavirus 2 (SARS-CoV-2). (A to C) HNO air-liquid interface (ALI) cells were apically infected with RSV/A/Ontario (ON) and RSVB/Buenos Aires (BA) at a multiplicity of infection (MOI) of 0.01. Apical and basolateral samples were collected at 1, 2, 5, and 10 days postinoculation (dpi) in two distinct HNO cell lines (HNO2 and HNO204). RNA was isolated from media, and copy numbers of RSV N gene RNA were determined using quantitative real-time PCR (qRT-PCR). Levels of RSV N genes (copies/mL) from (A) RSV/A/ON at different time points and (B) RSV/A/BA at different time points. (C) HNO-ALI cells were apically infected with SARS-CoV-2 at an MOI of 0.01. Apical and basolateral samples were collected at the time of infection and 3 and 6 dpi in three distinct HNO cell lines (HN02, HNO204, and HNO918). RNA was isolated from media, and SARS-CoV-2 copy numbers of N gene RNA were determined using qRT-PCR. (D and E) Infectious viral titers reported as PFU per mL for RSV/A/ON and RSV/B/BA using a quantitative plaque assay. Data shown were from two individual experiments with two technical replicates per group in each experiment and are represented as the mean ± standard deviation (SD).

We tested the ability of the HNO-ALI system to model RSV and SARS-CoV-2 viral infections using three HNO lines, HNO2, HNO204, and HNO918. The HNO-ALI system was apically inoculated with two contemporaneous RSV strains, RSV/A/ON and RSV/B/BA, representing RSV/A and RSV/B subtypes. RSV showed robust replication in both the HNO-ALI lines, reaching ∼5 × 10^7^ RNA copies/mL in the apical compartment by day 5 and reaching steady state over 10 days postinoculation (dpi) (Fig. 2A and B). The infection of HNO-ALI produced infectious virions as demonstrated by plaque assays on 2, 5, and 10 dpi (Fig. 2D and E and Fig. S4). Live infectious RSV was detected only on the apical side of the HNO-ALI and not on the basolateral compartment, consistent with RSV being predominantly released from the apical cells to the lumen of the HNO-ALI culture. The infection of HNO-ALI with SARS-CoV-2 (WA-1) also showed increases in extracellular viral loads reaching ∼5 × 10^8^ RNA copies/mL by day 3 and a plateau at 6 dpi (Fig. 2C). In contrast to RSV, the SARS-CoV-2 genome was detected on both the apical lumen and the basolateral compartment of the HNO-ALI system, suggesting SARS-CoV-2 virions are infecting cells in the apical layer and basolateral layers of the HNO epithelium later into the infection.

10.1128/mbio.03511-21.4 FIG S4 Infection of human nose organoid 918 air-liquid interface (HNO-918-ALI) culture with respiratory syncytial virus (RSV). (A and B) HNO-ALI cells were apically infected with RSV/A/Ontario (ON) and RSVB/Buenos Aires (BA) at a multiplicity of infection of 0.01. Apical and basolateral samples were collected at 1, 2, 4, and 8 days postinoculation (dpi). RNA was isolated from media, and copy numbers of RSV N gene RNA were determined using quantitative real-time PCR (qRT-PCR). Levels of RSV N gene (copies/mL) from (A) RSV/A/ON at different time points and (B) RSV/A/BA at different time points. (C and D) Infectious viral titers reported as PFU per mL for RSV/A/ON and RSV/B/BA using a quantitative plaque assay. The data shown were from two technical replicates per group and are represented as the mean ± SD. Download FIG S4, PDF file, 0.02 MB. Copyright © 2022 Rajan et al. 2022 Rajan et al. https://creativecommons.org/licenses/by/4.0/ This content is distributed under the terms of the Creative Commons Attribution 4.0 International license.

### Morphological analysis of RSV- and SARS-CoV-2-infected HNO-ALI systems.

**FIG 3:**
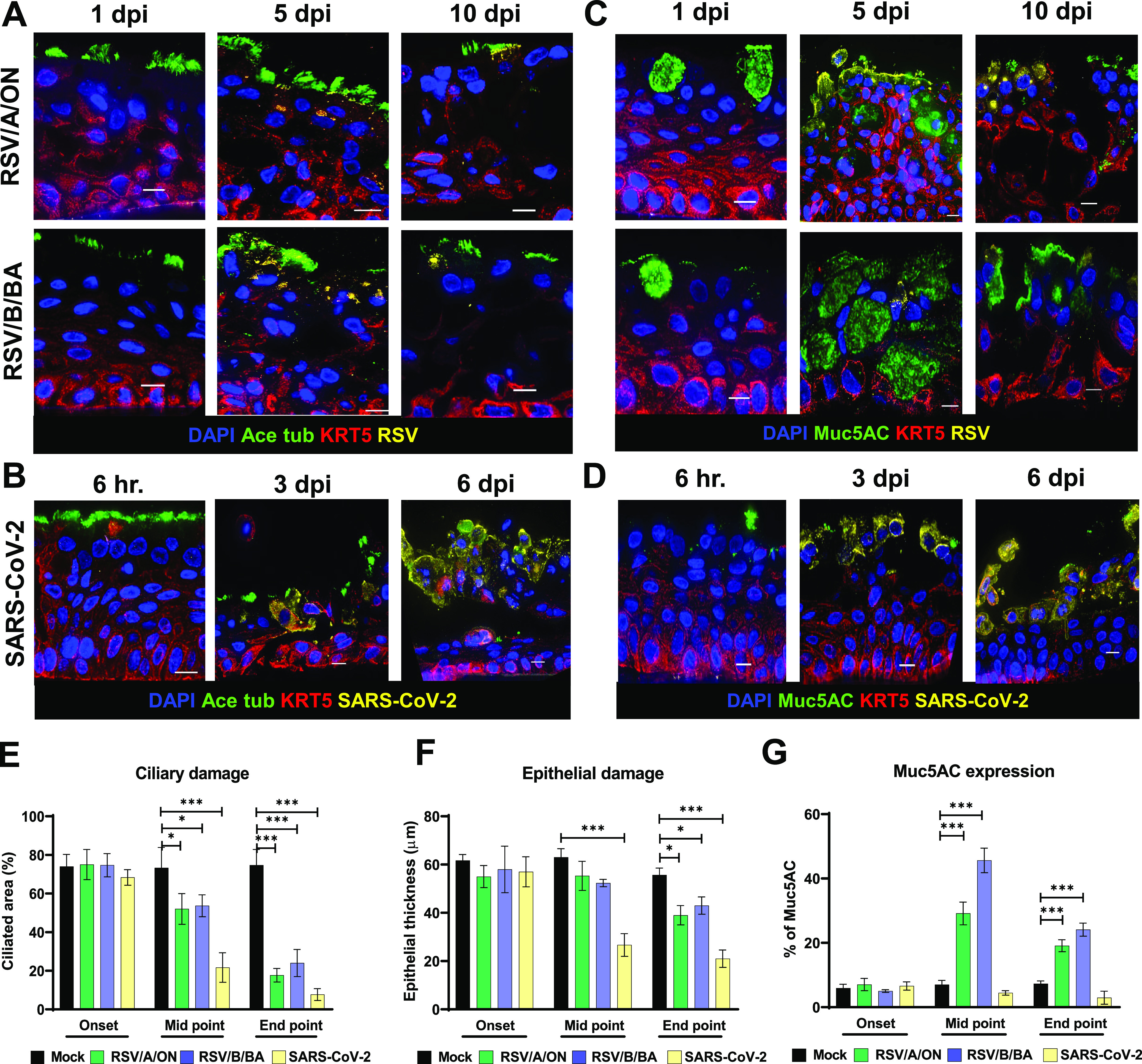
Immunofluorescence and morphological analysis of respiratory syncytial virus (RSV) and severe acute respiratory syndrome coronavirus 2 (SARS-CoV-2)-infected human nose organoid air-liquid interface (HNO-ALI) system. (A to D) Representative deconvoluted epifluorescence images of HNO cells showing nuclei (DAPI, blue) and basal cells (keratin 5 [KRT5], red). In panel A cilia (acetylated alpha tubulin [ace-tubulin], green) and RSV (F-protein, yellow) are shown; (B) cilia (ace-tubulin, green) and SARS-CoV-2 (spike protein, yellow); (C) goblet cells (mucin 5AC [MUC5AC], green) and RSV (fusion protein, yellow); (D) goblet cells (MUC5AC, green) and SARS-CoV-2 (spike protein, yellow). (E to G) Quantification of (E) ciliary damage, (F) epithelial damage, and (G) MUC5AC expression of HNO-ALI infected by RSV/A/ON, RSV/B/BA, and SARS-CoV-2. Data were collected from 10 representative images per group in each experiment and are represented as the mean ± SD. (*, *P* < 0.05; **, *P* < 0.01; ***, *P* < 0.001). Scale bar equals 10 μm.

Changes in epithelial cell morphology upon RSV and SARS-CoV-2 infection were examined and compared by immunofluorescence labeling of HNO-ALI cultures. Both RSV and SARS-CoV-2 infected HNO-ALI cultures recapitulated *in vivo* data, including apical shedding of virus-infected cells, ciliary damage, and epithelial thinning (Fig. 3A to D). Early infection (6 h and 1 dpi) was comparable between RSV/A/ON, RSV/B/BA, and SARS-CoV-2, but SARS-CoV-2 infection caused more damage at later time points (6 dpi). Also, RSV infection appeared confined to the apical cell layer. In contrast, SARS-CoV-2 spike protein antigen was detected deeper in the basal cell layer. Using fluorescence threshold analysis to quantify cilium expression (acetylated tubulin or Ace-Tub), we found that SARS-CoV-2 caused significantly higher ciliary damage in comparison to RSV (Fig. 3E). The thickness of the epithelium also was significantly lower in SARS-CoV-2 infected cells in comparison to RSV (Fig. 3F). The hematoxylin and eosin (H&E) and periodic acid-Schiff-alcian blue (PAS-AB) staining of HNO-ALI further revealed severe extrusion, epithelial thinning, and rounding of apical cells in SARS-CoV-2-infected samples (Fig. S2). In contrast, RSV but not SARS-CoV-2 showed hypersecretion of mucus as quantified by expression of MUC5AC marker (Fig. 3C, D and G).

10.1128/mbio.03511-21.2 FIG S2 Histological characterization of human nose organoids (HNOs) infected with the following respiratory viruses: RSV/A/Ontario (ON), RSV/B/Buenos Aires (BA), and severe acute respiratory syndrome coronavirus-2 (SARS-CoV-2). HNO air-liquid interface (ALI) cells were apically infected with RSV/A/Ontario (ON), RSVB/Buenos Aires (BA), or severe acute respiratory syndrome coronavirus 2 (SARS-CoV-2) at a multiplicity of infection (MOI) of 0.01. Hematoxylin and eosin (H&E) and periodic acid-Schiff-alcian blue (PAS/AB) staining were performed on HNO-ALI cultures at 10 and 6 days postinoculation (dpi) for RSV and SARS-CoV-2, respectively. Scale bar equals 50 μm FIG S2, PDF file, 0.2 MB. Copyright © 2022 Rajan et al. 2022 Rajan et al. https://creativecommons.org/licenses/by/4.0/ This content is distributed under the terms of the Creative Commons Attribution 4.0 International license.

### RSV but not SARS-CoV-2 predominantly induces interferon lambda (IFN-λ1) and IFN-γ inducible chemokine response in HNO-ALI cultures.

Characterizing the host immune response and identification of biomarkers is crucial for modeling of RSV and SARS-CoV-2 pathogenesis in these advanced HNO-ALI culture systems. To do this, we performed Luminex cytokine analysis of 29 cytokines/chemokines in HNO2-ALI (Data Set S3). Bronchial and nasal epithelial cells are known to secrete inflammatory cytokines in response to viral infections. We analyzed the levels of inflammatory cytokines induced by HNO-ALI cultures in response to (i) both RSV/A/ON and RSV/B/BA infection at 1 dpi, 2 dpi, 5 dpi, and 10 dpi and (ii) SARS-CoV-2 infection at 6 h, 3 dpi, and 6 dpi. In general, cytokine/chemokine levels peaked at either 5 dpi or 10 dpi for RSV-infected HNO-ALI cultures and at 3 dpi or 6 dpi for SARS-CoV-2-infected HNO-ALI cultures. Correlation analyses for these time-series experiments were not reported because they were not designed to determine an association between cytokine/chemokine concentrations and virus quantity at any one time.

10.1128/mbio.03511-21.9 DATA SET S3 Cytokine expression profile of HNO-ALI infected with RSV and SARS-CoV-2. Download Data Set S3, XLSX file, 0.04 MB. Copyright © 2022 Rajan et al. 2022 Rajan et al. https://creativecommons.org/licenses/by/4.0/ This content is distributed under the terms of the Creative Commons Attribution 4.0 International license.

**FIG 4:**
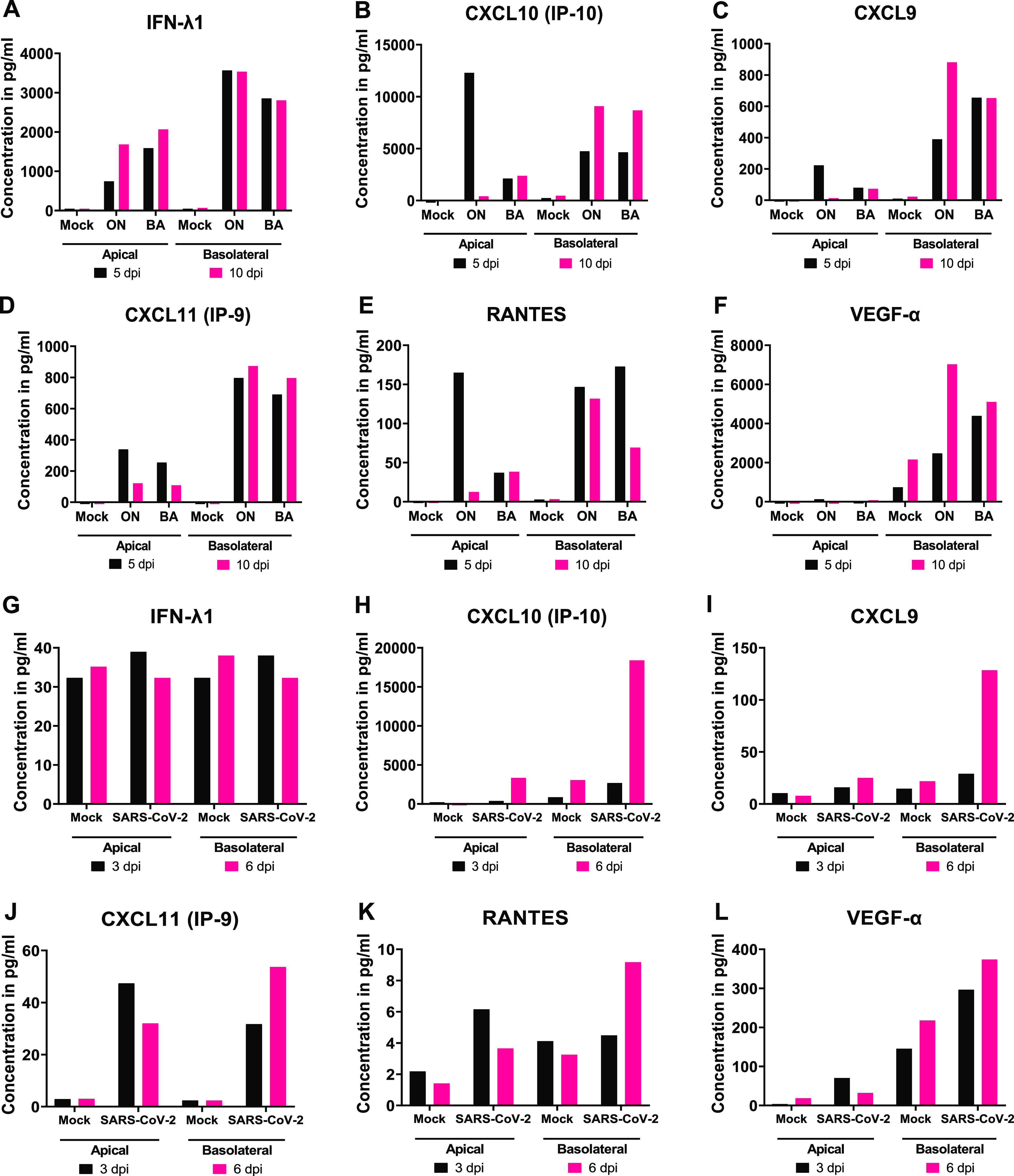
Immune cytokine/chemokine profile of human nose organoids (HNOs) infected with respiratory syncytial virus (RSV) and severe acute respiratory syndrome coronavirus 2 (SARS-CoV-2). HNO air-liquid interface (HNO-ALI) cultures were infected apically with RSV/A/Ontario (ON), RSVB/Buenos Aires (BA), and SARS-CoV-2 at a multiplicity of infection (MOI) of 0.01. The cultured supernatants from both the apical and basolateral compartments were harvested from mock-, RSV-, and SARS-CoV-2-infected HNO-ALI cultures. Profiles of extracellular cytokines and chemokines released in the apical and basolateral compartments were determined by the multiplex-Luminex cytokine assay. (A to F) Levels of cytokines and chemokines released from HNO2 cells infected with contemporaneous RSV strains RSV/A/ON and RSV/B/BA. (G and L) Levels of cytokines and chemokines released from HNO2 cells infected with SARS-CoV-2 (WA-1 strain). The data shown were from two technical replicates per group, and the Luminex assay was run with each sample in duplicate and assessed for cytokines.

RSV infection induced a strong IFN-λ1/interleukin-29 (IL-29) response in HNO-ALI cultures at 5 dpi and 10 dpi (Data Set S3). In striking contrast, HNO2-ALI showed no changes in the levels of IFN-λ1/IL-29 in response to SARS-CoV-2 infection (Fig. 4A and G). Notably, for RSV, strong inductions were observed for chemokine (C-X-C motif) ligand 10 (IP-10), CXCL9, and CXCL11/IP-9 and regulated upon activation, normal T cell expressed and secreted (RANTES) from both the apical and basolateral side of the Transwells (Fig. 4B–4E). The C-X-C chemokine ligands are generally upregulated by IFN-gamma (IFN-γ) produced from activated T cells and natural killer cells, none of which were present in the HNO-ALI cultures. Next, interleukin-8 (IL-8), a classical biomarker of RSV infection, was also detected at 5 dpi and 10 dpi at both the apical and basolateral sides of HNO-ALI cultures (Data Set S3) (16). RSV induced high levels of vascular endothelial growth factor A (VEGF-α), specifically on the basolateral side of HNO-ALI. SARS-CoV-2 infection showed similar trends in the levels of the same cytokines, but the concentration of these cytokines was much lower than those for RSV (Fig. 4I to L). In contrast, an increase in CXCL10 (IP-10) by ∼100-fold was measured on the basolateral side of SARS-CoV-2-infected samples at 6 dpi, which has been reported as a biomarker of COVID-19 disease severity (Fig. 4H) (17).

An increase in matrix metalloproteinase 9 (MMP-9) was reported in COVID-19 patients with respiratory failure (18) and during RSV infection (19). However, we did not detect significant changes in the levels of MMP-9 or MMP-7 to either RSV or SARS-CoV-2 (Data Set S3). Perhaps this could be due to the inhibitory effect of tissue inhibitors of metalloproteinase 1 (TIMP-1) on MMPs. Additionally, for both RSV and SARS-CoV-2, we did not detect any changes in levels of some pulmonary fibrosis biomarkers, such as transforming growth factor beta (TGF-β), fibroblast growth factor (FGF), granulocyte colony-stimulating factor (G-CSF), and granulocyte-macrophage colony-stimulating factor (GM-CSF) (20). On the other hand, VEGF was elevated and has been associated with disease severity in idiopathic pulmonary fibrosis (20). Though measured in small amounts (less than 100 pg/mL), we noticed an increase in levels of proinflammatory immune mediators such as IL-6, and IL-1α for both RSV and SARS-CoV-2 as seen in clinical disease (Data Set S3). In contrast, only for RSV and at the basolateral side, monocyte chemoattractant protein 1 (MCP-1), macrophage inflammatory protein-1 beta (MIP-1β), and tumor necrosis factor-α (TNF-α) levels increased at 5 dpi. Eotaxin, IL-1β, and MCP-3 were both increased at the apical and basolateral sides at low levels in response to RSV infection (Data Set S3).

### *Ex vivo* human challenge respiratory virus model in the HNO-ALI system.

Although multiple preclinical animal models are available to recapitulate some pathognomonic aspects of RSV and SARS-CoV-2 infection, they do not faithfully represent the physiology of human airway epithelium (21–26). Additionally, there are advanced iPS cell- and lung tissue-derived AO models where the disease can be modeled, but they require access to clinical samples to establish these culture systems (7, 27, 28). Thus, there is an unmet and urgent need for physiologically relevant yet easily accessible preclinical human airway models for respiratory viral diseases to test the efficacy of therapeutics. To address this, we tested the ability of the HNO-ALI system to act as an *ex vivo* human challenge respiratory virus model to test the efficacy of a known therapeutic monoclonal antibody (MAb) to RSV infection. Palivizumab (Synagis) is a neutralizing MAb targeted against the F-glycoprotein of RSV that prevents RSV-cell fusion and hence reduces RSV replication (29). We introduced the palivizumab MAb in the basolateral compartment and monitored its neutralizing capacity on the apical lumen, mimicking the neutralizing effects of MAb in circulation on the virus-exposed airway epithelium (Fig. S3B). The palivizumab concentrations used were in the biological range shortly after intravenous injection (640 μg/mL) or prior to the next administration dose (80 μg/mL) (30). The translocation of palivizumab to the apical side was measured using an antibody kinetics assay as shown in Fig. S5. Approximately 5% of the concentration of palivizumab in the basolateral compartment was detected in the apical lumen in both uninfected and RSV-infected HNO-ALI cultures.

10.1128/mbio.03511-21.3 FIG S3 Human nose organoid air-liquid interface (HNO-ALI) infection and airway challenge model. Download FIG S3, PDF file, 0.2 MB. Copyright © 2022 Rajan et al. 2022 Rajan et al. https://creativecommons.org/licenses/by/4.0/ This content is distributed under the terms of the Creative Commons Attribution 4.0 International license.

10.1128/mbio.03511-21.5 FIG S5 Palivizumab kinetics assay. The antibody kinetics assay demonstrates the palivizumab antibody translocation from the basolateral compartment to the apical compartment in human nose organoid air-liquid interface (HNO-ALI) culture. HNO-ALI cells were apically infected with RSV/A/Ontario (ON) at a multiplicity of infection of 0.01. Apical and basolateral samples were collected at 1, 2, 4, and 8 days postinoculation (dpi) and measured for palivizumab by a palivizumab competitive antibody assay. The lower limit of detection was 1 μg/mL. The data shown were from two technical replicates per group and are represented as the mean ± SD. Download FIG S5, PDF file, 0.01 MB. Copyright © 2022 Rajan et al. 2022 Rajan et al. https://creativecommons.org/licenses/by/4.0/ This content is distributed under the terms of the Creative Commons Attribution 4.0 International license.

**FIG 5:**
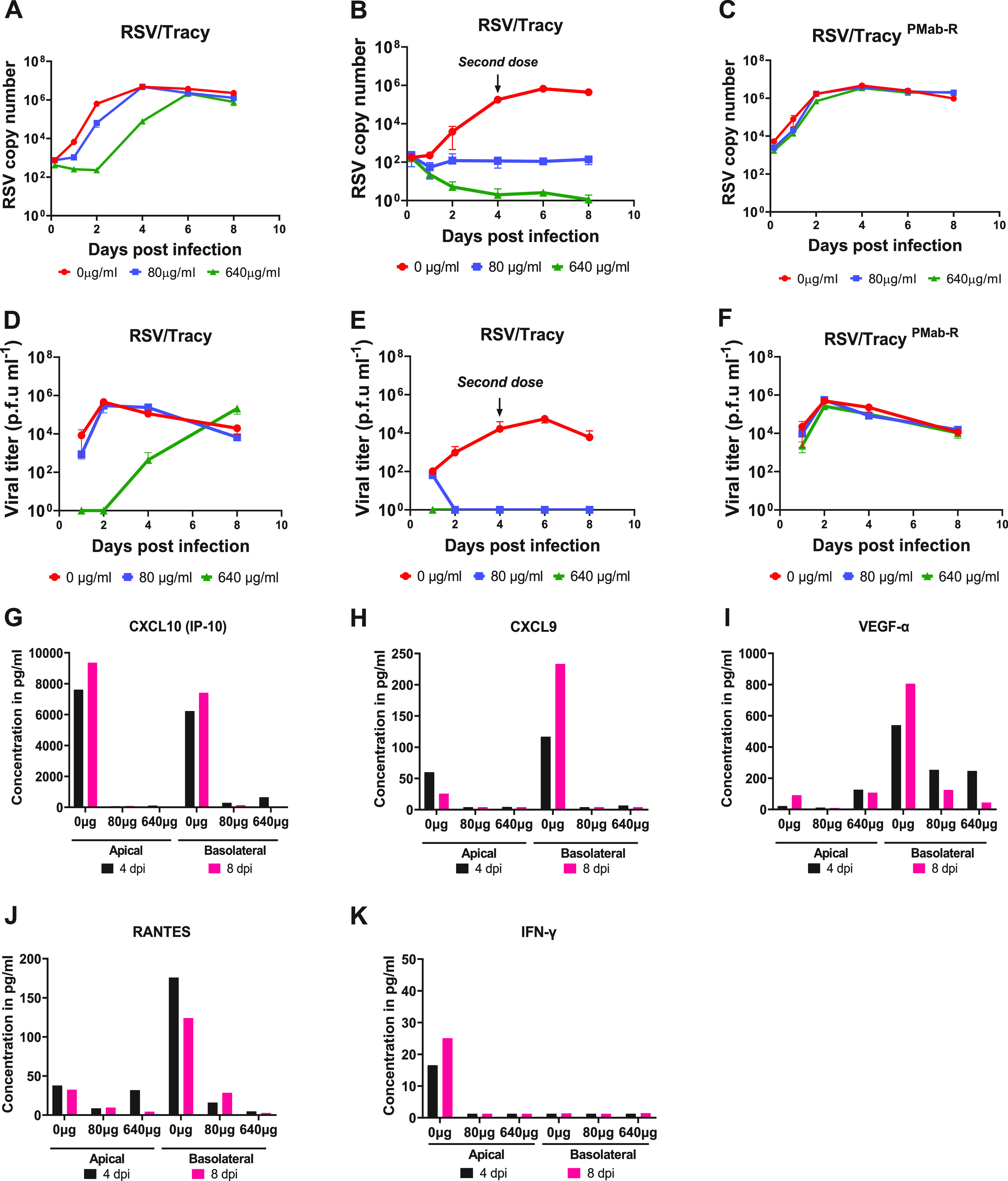
Immunoprophylaxis treatment for respiratory syncytial virus (RSV) infection in human nose organoids (HNOs). (A to C) Viral copy numbers of RSV/A/Tracy after treatment with palivizumab of 0 μg/mL, 80 μg/mL, and 640 μg/mL at 1, 2, 4, 6, and 8 dpi were measured. Panel A demonstrates only an initial but not sustained decrease in RSV copy number with a short exposure of palivizumab pretreatment. Panel B shows a significant reduction in RSV replication during the long exposure of palivizumab across the infection period in a dose-dependent manner with the addition of a second dose of palivizumab at day 4. Panel C exhibits specificity of palivizumab pretreatment, as strain RSV/A/Tracy^P-Mab-R^ is resistant to the antibody and hence showed no decrease in infection. (D to F) Quantification of infective viral particles using a plaque assay for the experiments described above. The data shown were gathered from two technical replicates per group in each experiment and are represented as the mean ± SD. (G to K) The levels of inflammatory cytokines released by RSV-infected HNOs under palivizumab treatment conditions.

HNO2-ALI cultures preincubated with palivizumab at 640 μg/mL for 90 min suppressed RSV/A/Tracy replication up until 2 dpi. Thereafter, RSV replication resumed at 4 dpi, although at reduced levels compared to the no-palivizumab control, and replication reached a peak at 6 dpi and finally plateaued at 8 dpi (Fig. 5A). This both demonstrated the efficacy of a short exposure of palivizumab to reduce infection in the apical ciliated cells and also suggested a decline in bioavailability of palivizumab to persistently prevent RSV replication at later time points. In a subsequent experiment, HNO-ALI cultures were preincubated with palivizumab prior to the virus inoculation and maintained throughout the duration of infection (long exposure). The cells were refed on the basolateral compartment 4 dpi using the same palivizumab concentration (second dose). In this long-exposure experimental setup, RSV/A/Tracy failed to replicate in HNO-ALI culture at both 640 μg/mL and 80 μg/mL of palivizumab (Fig. 5B). Nonetheless, palivizumab-resistant strain RSV/Tracy^P-Mab R^ (31) readily replicated in palivizumab-pretreated HNO-ALI cultures, demonstrating the specificity of palivizumab in preventing RSV infection (Fig. 5C). Furthermore, we analyzed the cytokines expressed by palivizumab-preincubated-HNOs that were inoculated with RSV. Long exposure of palivizumab with either the 80 or 640 μg/mL dose not only prevented RSV replication (Fig. 5B and E) but also abolished the virus-induced inflammatory cytokines that would have been released into the apical lumen and basolateral compartment (Fig. 5G to K).

## DISCUSSION

Airway organoids are three-dimensional airway culture systems that were first developed in 1993 with self-organizing 3D structures (32). To date, there are only a few AO models that are produced using invasive lung biopsy or bronchoalveolar lavage (BAL) fluid patient-donor materials (4, 6, 7, 9, 33–38). In this study, for the first time, we describe a noninvasive, reproducible, and reliable approach to establish human nose organoids (HNOs) that allows for long-term expansion. Earlier studies used invasive nasal brushing samples or biopsy samples that are traditionally obtained from human subjects undergoing bronchoscopy procedures. In our protocol, we used human nasal wash and midturbinate swab samples from healthy volunteers to generate nasal organoids. The ease in obtaining the nasal wash/midturbinate swab samples facilitates our noninvasive approach in the general adult population as well as the vulnerable pediatric population. These nasal wash and midturbinate samples can either be self-collected or collected by a trained laboratory technician. A notable difference between our HNO-ALI culture and other ALIs is the richness and stratification of the airway epithelium (8, 35–38). While nasal epithelial cells reported by Müller et al. and Brewington et al. (8, 38) can be grown in ALI conditions, they are predominantly represented as a single monolayer of cells. In contrast, our HNO-ALI system derived from HNOs exhibits a complex pseudostratified epithelium interlaced with basal, goblet, club, and ciliated cells with spontaneous synchronization of beating cilia (Fig. 1D to G). In short, this newly developed HNO model offers an elegant solution to develop *in vitro* human respiratory airway models that can be used across several basic-science laboratories to model respiratory diseases.

RSV infections cause millions of hospitalizations every year (10), and almost all children are infected with RSV by 2 years of age, with repeated reinfections occurring throughout life (13). Another important pathogen in the current scenario is SARS-CoV-2, the causative organism of COVID-19. AO systems are known for the robustness to model respiratory viral infections such as SARS-CoV-2 (9, 39), influenza virus (34), enterovirus (40), and other respiratory diseases (1). We tested the usefulness of our HNOs as an *ex vivo* culture system for studying two major human respiratory viruses—RSV and SARS-CoV-2. In traditional cell culture models, including immortalized and primary airway epithelial cell lines, the viral inoculum is introduced in the cell culture medium. This limits the exposure of air to the airway epithelium, which is a critical component for physiological relevance. Additionally, most of the existing 3D airway organoid models have the apical side facing inward, and microinjections are needed to establish infection. In our HNO-ALI model, the apical side of the epithelium is both accessible and air-exposed, thus making it both physiologically relevant and highly suitable for studying respiratory viral infections. Here, we report that the HNO-ALI system readily supports the replication and growth of RSV and SARS-CoV-2 (Fig. 2A to C), and the newly released virions from these cultures are infectious (Fig. 2D and E). Using immunohistochemistry, we also showed that the apical ciliated epithelial cells are indeed the main target for RSV (Fig. 3A). These results are consistent with previously published studies on AOs and RSV where apical infection of ciliated cells was observed (7, 41–43). Although we did not detect any RSV infection of basal cells, in contrast to our observation, a previous study has noted that basal cells if exposed to RSV are susceptible to RSV infection, suggesting a pathogenic mechanism for additional airway damage in individuals with chronic lung disease (44). Next, in our study, we observed that only RSV induced a robust increase in expression of MUC5AC (Fig. 3C), and this was comparable to the Persson et al. study, where they have also reported expansion of MUC5AC secretory cells in response to RSV infection (44). Unlike RSV, SARS-CoV-2 induced severe damage to the ciliary epithelium without an increase in mucus secretion (Fig. 3B and D).

We performed a thorough characterization of the host immune response to RSV and SARS-CoV-2 infection in the HNO-ALI system. Although RSV infection predominantly occurred at the apical surface, we measured higher levels of cytokine response to viral infection on the basolateral side, suggesting translocation of infection signals from the apical side to the basolateral compartment mimicking physiological relevance. We also observed a strong type-III interferon (IFN-λ1/IL-29) response for RSV and not SARS-CoV-2, suggesting stark differences in the role of the epithelial cells in initiating the early innate and antiviral immune signaling. It was interesting to observe that the increase in IFN-λ1/IL-29 responses to RSV at 5 dpi was associated with a reduced or stalled viral replication beyond 5 dpi as noted by plateauing of viral copy number measured by real-time PCR (RT-PCR) (Fig. 2A and B and 4A) or infectious virus (Fig. 2D and E and 4D) measured by a plaque assay. Additionally, a previous study has shown an association of IFN-λ1/IL-29 and an increase in MUC5AC secretory cells during RSV infection (44). Consistent with these findings, our data also demonstrate an increase in both IFN-λ1 and MUC5AC expression in response to RSV infection in HNO-ALI cultures (Fig. 3B and D and 4A). However, future studies are needed to clarify the mechanisms of hypersecretion of MUC5AC induced by RSV in the HNO-ALI system.

An IFN-γ-inducible cytokine/chemokine response (IP-10, CXCL-9 and RANTES) and increase in VEGF-α was noted for both RSV and SARS-CoV-2 infection (Fig. 4). These data are in accordance with clinical findings in patients from previously published studies, where high levels of IFN-γ and CXCL10 (IP-10) were seen for RSV (45–47). IP-10, a biomarker for both RSV and COVID-19, severity was detected in the infected HNO2 cultures. In contrast, a study conducted by Rijsbergen et al. showed no increase in IP-10 in response to RSV infection in nasal epithelial cells (43). It is important to note that previous studies using airway epithelial culture systems have shown and supported the growth of RSV and SARS-CoV-2 only up to 4 dpi (7, 28, 39). However, in our system, we extended the viral infection for RSV up to 10 days and for SARS-CoV-2 up to 6 days. This allows for long-term study of viral infections. Thus, we hope that this HNO-derived airway epithelial model will greatly enhance the understanding of RSV and SARS-CoV-2 pathogenesis.

Currently, there is an unmet need for an easily available preclinical human model to study RSV and SARS-CoV-2 infections that recapitulates the human experience. While animal models of infection have provided major insights, they have also misdirected our understanding of viral pathogenesis and prevention (25). Also, the cost, safety, and ethical issues associated with human challenge models limit their use and access (48). Our *ex vivo* HNO-ALI model is an alternative to the human challenge model. In our study, HNO-ALI cultures were pretreated with palivizumab to model immunoprophylaxis treatment to prevent RSV infection. A single dose and short-exposure to palivizumab of HNO-ALI culture was only partially effective in reducing RSV replication (1 dpi at 80 μg/mL and up to 2 dpi at 640 μg/mL). Nonetheless, when an immunoprophylaxis-exposure strategy of palivizumab was utilized, RSV was unable to replicate in the HNO-ALI system, and the HNOs did not produce inflammatory cytokines in response to RSV infection, demonstrating the immunoprophylactic protection against RSV infection and disease. Indeed, our HNO-ALI system more closely resembled the human experience where therapeutic MAb or polyclonal antibodies are administered intramuscularly or intravenously, respectively, to get into the blood circulation and provide protection of the airways against RSV infection (49–51). In our model, it was unclear if palivizumab neutralized the virus on the apical lumen or if virus neutralization occurred intracellularly. Future studies will allow us to determine the mechanism of immune protection using HNO-ALI cultures. Thus, these HNOs will provide a more precise human milieu and can function as a preclinical human model to investigate promising therapeutics while recapitulating the complex interactions between the drug, the virus, and the airway cells.

We are of the opinion that, at the current stage, the HNO-ALI system remains at a highly reductionist level and has the potential for improvements. This includes advancements of the HNO-ALI system with (i) addition of immune cells, (ii) endothelial cells to further mimic the complex physiology of organs; (iii) furthermore, genetic knock-ins and knockouts can be made to study the role of specific host components in microbial infections and human diseases. With these future advancements, the HNOs can be optimized to develop next-generation *in vivo* human airway models and used as a valuable tool to evaluate pathogenesis, therapeutics, and vaccine candidates for major global respiratory viral pathogens. In addition, the HNOs retain the genetic background of the individual, thus allowing the possibility to screen drugs for cancer therapeutics, genetic-disease modeling, and development of personalized medicine.

## MATERIALS AND METHODS

### Obtaining nasal wash/nasal swab samples.

The samples were collected under the Institutional Review Board (IRB) of the Baylor College of Medicine (BCM), Houston, TX, USA, with written informed consent. Self-collected nasal wash samples were obtained by instilling 3 mL of saline into each nostril and collecting the fluid in a sterile cup. A paired midturbinate nasal swab sample from the same volunteer was also obtained using a flocked swab. The paired nasal wash and nasal swab samples were mixed and stored on ice until further processing. Inclusion of both nasal wash and midturbinate samples is necessary for optimal recovery of stem cells from the donors. Just the use of nasal wash samples does not yield viable nose organoids.

### Establishing human nose organoids (HNOs).

The generation of nasal wash- and nasal swab-derived HNOs was based on the published protocol (7). The nasal wash along with the flocked swab was spun at 80 × *g* for 5 min at 4°C. Supernatant was carefully removed and sheared using a 29-gauge insulin syringe to break up mucus (if any). Digestion medium (10 mL AO medium + 10 mg collagenase [Sigma C9407] + 100 μL amphotericin B [Fungizone]) was added, and the falcon tube was kept on a rocker for 30 to 60 min at 37°C. After digestion, the nasal swab was discarded, and fetal bovine serum (FBS) was added to inactivate collagenase. The above-described solution was sheared using a syringe, strained through a 100-μm strainer, and spun at 80 × *g* for 5 min at 4°C. The supernatant was removed, and the cell pellet was washed twice with wash medium (96 mL advanced Dulbecco’s modified Eagle’s medium [DMEM]/F12 + 1 mL GlutaMAX 100× + 1 mL HEPES 1 M + 1 mL Pen/Strep + 1 mL amphotericin B) and spun at 80 × *g* for 5 min at 4°C. Finally, the wash medium was removed, and the cell pellet was suspended in Matrigel and plated onto a 24-well plate and incubated for 10 min at 37°C. Once the Matrigel had solidified, 500 μL of AO medium with penicillin-streptomycin-amphotericin (PSA) was added to each well, and the plate was transferred into 37°C incubator. AO medium was replaced every 4 days and passaged every other week at a 1:2 ratio (wells) for expansion.

### Generation of HNO air-liquid interface (ALI) cultures.

The mature 3D HNOs were enzymatically and mechanically sheared to make ALI cultures using our previous method adopted from enteroid monolayer technology and conditions utilized for growing human bronchial epithelial cells (33, 52–55). Clear Transwells (Corning Costar, catalog no. 3470) were precoated with 100 μL of bovine type I collagen at 30 μg/mL (Gibco, catalog no. A1064401) and placed in an incubator for 1.5 h at 37°C. HNOs cultured in AO medium for 7 days were dissociated using 0.5 mM EDTA and spun at 300 × *g* for 5 min at 4°C. Single cells were obtained by adding 0.05% trypsin/0.5 mM EDTA (Invitrogen, catalog no. 25300054) for 4 min at 37°C. Trypsin was inactivated by addition of AO medium containing 10% FBS. The HNOs were dissociated vigorously using pipette tips, passed through a 40-μm strainer (Falcon, catalog no. 352340), and pelleted at 400 × *g* at room temperature for 5 min to generate single cells. The pellet was resuspended in AO medium containing 10 μM Y-27632 + epidermal growth factor (EGF) (Peprotech-AF-100-15). The collagen coating from the Transwells was removed and washed with phosphate-buffered saline (PBS), and the single cells were added at a seeding density of 3 × 10^5^ cells/well. Then, 750 μL of AO medium + EGF containing 10 μM Y-27632 (Sigma, catalog no. Y-0503) was added into the lower compartment of the Transwells. After 4 days, confluent monolayers were cultured under air-exposed conditions using differentiation medium (PneumaCult-ALI medium from STEMCELL Technologies) in lower compartments of the Transwells until 21 days.

### Viral infection, PCR, and plaque assays.

An overview of the infection method is provided in Fig. S3A. The differentiated HNO-ALI cells were apically infected with RSV/A/USA/BCM813013/2013(ON) (RSV/A/ON), RSV/B/USA/BCM80171/2010(BA) (RSV/B/BA), and SARS-CoV-2 (isolate USA-WA1/2020, obtained from Biodefense and Emerging Infectious resources [BEI]) at a multiplicity of infection (MOI) of 0.01 (30 μL/well). All work with SARS-CoV-2 was performed in a class II biosafety cabinet in the biosafety level 3 (BSL-3) high-containment facility at BCM. For mock infection, AO-differentiation medium (30 μL/well) alone was added. The inoculated plates were incubated for 1.5 h at 37°C with 5% CO_2_. At the end of the incubation, the inoculum was removed from the HNO-ALI system and left air-exposed for the defined period of infection. At the respective time points, the apical side of the Transwells was washed twice with 150 μL of AO differentiation medium and mixed with an equal volume of 15% glycerol Iscoves medium. On the basolateral side, 300 μL of the medium was removed and mixed with an equal volume of 15% glycerol Iscoves medium. The obtained samples were used for detection of viral RNA, infectious virions, and host cytokines. Samples were snap-frozen and stored at −80°C. The viral RNA was extracted using a mini viral RNA kit (Qiagen Sciences) in an automated QIAcube platform according to the manufacturer’s instructions (56). Viral RNA was detected using real-time PCR (RT-PCR) with primers targeting the nucleocapsid gene for RSV and SARS-CoV-2 as previously described (56, 57). RSV titer was measured using a semiquantitative plaque assay as previously described (58).

### Immunohistochemistry (IHC) and immunofluorescence labeling (IF).

HNO-ALI cultures were fixed in image-iT fixative solution (4% formaldehyde) (catalog no. FB002) for 15 min followed by dehydration in ethanol series (30%, 50%, 70%, and 90%, each 30 min at room temperature or overnight at 4°C). The Transwell membranes were then subjected to paraffin embedding and sectioning. Standard hematoxylin and eosin (H&E) and periodic acid-Schiff-alcian-blue (PAS/AB) staining were performed. For immunofluorescent labeling, the sections were deparaffinized in Histo-Clear (Electron Microscopy Science, catalog no. 64111-01), followed by washes in an alcohol sequence (100 > 100 > 95 > 65 %). The slides were then rehydrated and exposed for heat-induced antigen retrieval in 10 mM sodium citrate buffer pH 6 for 20 min at subboiling temperature (59). The sections were rinsed in water and blocked for 60 min in 1% bovine serum albumin (BSA) in Tris-buffered saline with Tween 20 (TBS-T) (blocking buffer). The sections were incubated overnight at 4°C with the following primary antibodies: 0.25 μg/mL keratin 5 (KRT5; BioLegend, catalog no. 905503), 0.2 μg/mL SCGB1A1 (CC10; Santa Cruz, catalog no. sc-365992 AF488), 0.2 μg/mL acetylated alpha tubulin (Santa Cruz, catalog no. sc-23950 AF488), 0.2 μg/mL mucin 5AC (Invitrogen, catalog no. 45M1), goat anti-RSV IgG antibody (Abcam, catalog no. ab20745), and rabbit anti-SARS-CoV-2 S1 IgG antibody (Sino Biologicals, catalog no. 40150R007; BEI Resources, catalog no. NR10361, 1:2,000 dilution of the antiserum). Primary antibodies were washed three times in TBS + 0.05% Tween for 10 min each, incubated with secondary antibodies from Invitrogen for 1 h at room temperature, washed twice with TBS-T, stained with 4′,6-diamidino-2-phenylindole (DAPI), washed twice with PBS, and mounted in VECTASHIELD Plus antifade mounting medium (Vector Laboratories, Burlingame, CA; H-1900). The slides were stored at 4°C until imaging.

### Immunofluorescence image quantification and analysis.

High-quality/high-resolution automated imaging was performed on a Cytiva DV live epifluorescence image restoration microscope using an Olympus Plan Apo N 60×/1.42 numerical aperture (NA) objective and a 1.9 k by 1.9 k pco.edge sCMOS_5.5 camera with a 1,024 by 1,024 field of view (FOV). The filter sets used were DAPI (390/18 excitation, 435/48 emission), FITC (475/28 excitation, 523/36 emission), TRITC (542/27 excitation, 594/45 emission), and CY5 (632/22 excitation, 676/34 emission). Z stacks (0.25 μm) of the whole section (∼10 μm) were acquired before applying a conservative restorative algorithm for quantitative image deconvolution using SoftWorx v7.0. Maximum intensity projections were used for image analysis and processed using ImageJ/Fiji. Between 6 and 10 slides were imaged per treatment/biological replicate and analyzed for IF studies. Every experiment was performed in two technical Transwell replicates and repeated for a minimum of two biological replicates. For visualization, RSV/SARS-CoV-2 spots were enhanced by histogram stretching across treatments, post-image acquisition in Fiji. The average area of ciliated epithelium was quantified by cell counts with acetylated-tubulin in Fiji at the beginning of each infection (day 1 for RSV, 6 h for SARS-CoV-2), the midpoint (day 5 for RSV, 3 days for SARS-CoV-2), and the endpoint of infection assays (day 10 for RSV and day 6 for SARS-CoV-2). Quantification of the amount of epithelial damage by RSV-A and SARS-CoV-2 was measured by calculating the average thickness of the epithelium in μm. The percentage of goblet cells (MUC5AC labeled area) relative to DAPI after infection with RSV-A, RSV-B, and SARS-CoV-2 was measured using the formula “μm^2^ MUC5AC label/μm^2^ of epithelium (DAPI)” at the beginning of each infection (day 1 for RSV, 6 h for SARS-CoV-2), the midpoint (day 5 for RSV, 3 days for SARS-CoV-2), and the endpoint of infection assays (day 10 for RSV and day 6 for SARS-CoV-2). GraphPad Prism 9.0 was used to construct graphs and perform statistical tests.

### Multiplex Luminex immunoassays.

Cytokines and chemokines secreted by HNO-ALI cells were measured and analyzed using the Milliplex cytokine/chemokine magnetic bead panel (Millipore) according to the manufacturer’s instructions. The kits used in this study include (i) the Milliplex human cytokine panel with eotaxin/CCL11, FGF-2, G-CSF, GM-CSF, IL-1a, IL-1b, IL-6, IL-8/CXCL8, IL-17E/IL-25, IP-10/CXCL10, MCP-1, MCP-3, MIG, MIP1a, MIP1b, RANTES/CCL5, TNFa, VEGF-A, IL-33, TRAIL, TSLP, TAC/CXCL11, IL-29, BAFF, and HMGB1, (ii) the TGFb1 Singleplex kit, (iii) the Milliplex human MMP panel 2 with MMP9 and MMP7, and (iv) the Milliplex human TIMP panel 2 with TIMP1. Data were obtained with Luminex xPONENT for MAGPIX v4.2 build 1324 and analyzed with MILLIPLEX Analyst v5.1.0.0 standard build 10/27/2012.

### Immunoprophylaxis model of HNO-ALI.

To establish and test the feasibility of HNO-ALI system to serve as an *ex vivo* human airway challenge model, we developed an immunoprophylaxis system using palivizumab antibodies to prevent RSV infection. An overview of the immunoprophylaxis model is summarized in Fig. S3B. The differentiated HNO-ALI cells were apically infected with RSV/A/USA/BCM-Tracy/1989 (GA1) and RSV-Tracy resistant to palivizumab at a multiplicity of infection of 0.01 (30 μL/well). In our model, palivizumab was introduced on the basolateral compartment either (i) 30 min prior to RSV infection and during the RSV incubation for a total of 90 min (short exposure) or (ii) 30 min prior to RSV infection and maintained throughout the duration of infection (long exposure). For the long exposure, at 4 dpi, the cells were refed on the basolateral side with medium containing the same palivizumab concentration (second dose). Palivizumab was administered on the basolateral side of the HNO-ALI system at 80 μg/mL and 640 μg/mL.

### Detection of palivizumab in HNO-ALI cultures.

To determine and quantify if palivizumab introduced in the basolateral compartment of HNO-ALI cultures can translocate across the membrane and neutralize RSV, we introduced 80 μg/mL palivizumab to the basolateral compartment of HNO-ALI cultures 30 min prior to infection with RSV/A/USA/BCM-Tracy/1989 (GA1) at an MOI of 0.01 (30 μL/well) or mock infection. At predetermined time points, the apical and basolateral compartments were sampled, and palivizumab concentrations were analyzed using the palivizumab competitive antibody assay (PCA) as previously described (60). In brief, a standard curve of palivizumab (2-fold serial dilution from 12,500 ng/mL to 24.41 ng/mL) was generated. Next, 50 μL of 2-fold serial dilutions of test sample (1:5 to 1:2,560) in duplicate were added to the fusion protein-coated plates, followed by 50 μL of 100 ng/mL biotinylated palivizumab and a 1-h incubation. After washing, horseradish peroxidase (HRP)-conjugated streptavidin (SeraCare Life Sciences, Gaithersburg, MD) was added for an additional hour. Wells containing biotinylated palivizumab without test sample served as positive controls representing maximum binding. Wells containing 5% milk instead of test sample and wells containing test sample without biotinylated palivizumab served as negative controls. A four-parameter logistic (4PL) regression model was used to calculate the PLA concentrations (μg/mL) based on the dynamic range of the standard curve by interpolating the concentration of the standards that correspond to the absorbance value at which the test sample resulted in 50% inhibition. The lower limit of detection (LLoD) was 1 μg/mL. The palivizumab kinetics assay was performed for the immunoprophylaxis-exposure model.

### Statistical analysis.

Statistical significance was determined by two-way analysis of variance (ANOVA). Tukey’s multiple-comparison tests were performed using Prism v7.0 for Windows (GraphPad Software, San Diego, CA). Differences in the means are considered significant at *P* ≤ 0.05 with specific *P* values detailed in the figure legends.

10.1128/mbio.03511-21.6 TABLE S1 Airway organoid (AO) medium composition. Download Table S1, XLSX file, 0.01 MB. Copyright © 2022 Rajan et al. 2022 Rajan et al. https://creativecommons.org/licenses/by/4.0/ This content is distributed under the terms of the Creative Commons Attribution 4.0 International license.
