# Supplementary figures and images for: The Human Nose Organoid Respiratory Virus Model: an Ex Vivo Human Challenge Model To Study Respiratory Syncytial Virus (RSV) and Severe Acute Respiratory Syndrome Coronavirus 2 (SARS-CoV-2) Pathogenesis and Evaluate Therapeutics
Source: mBio. 2022 Feb 15;13(1):e03511-21. doi: 10.1128/mbio.03511-21 (PMC8844923; doi:10.1128/mbio.03511-21)

## Ciliary epithelium associated genes

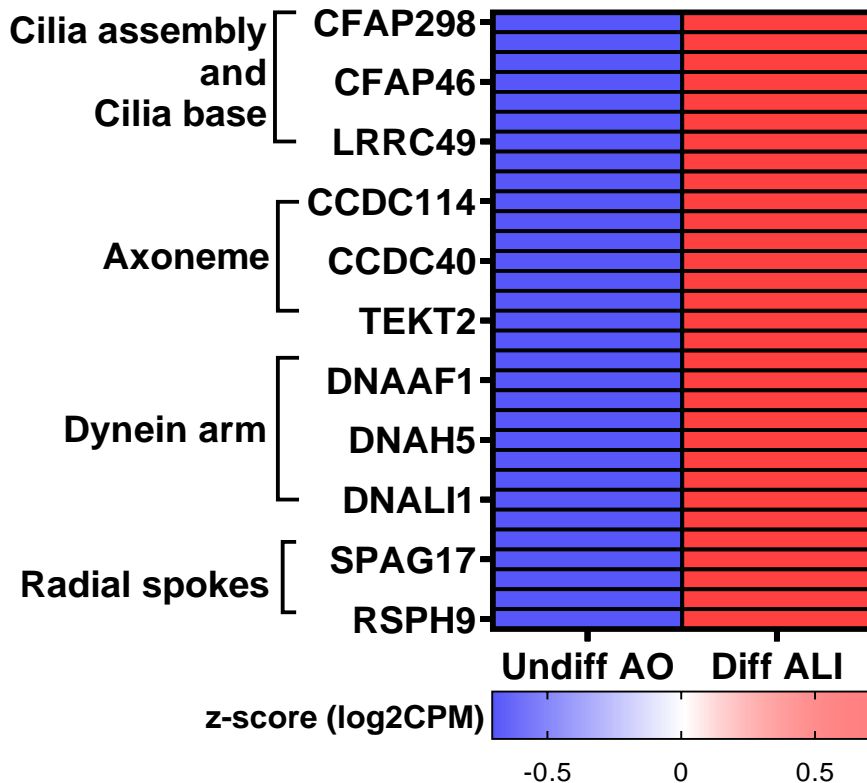

Supplement: FIG S1 [file mbio.03511-21-sf001.pdf]

**A****RSV/A/ON**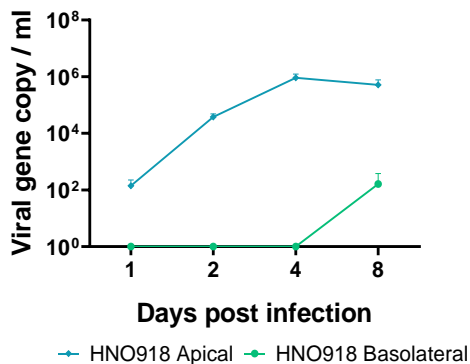**B****RSV/B/BA**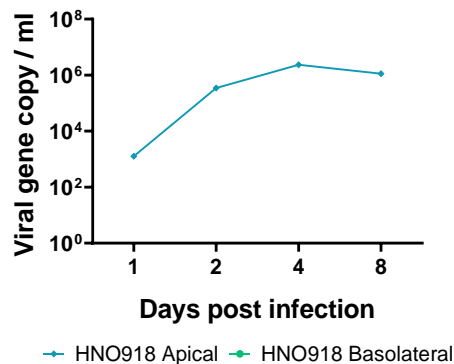**C****RSV/A/ON**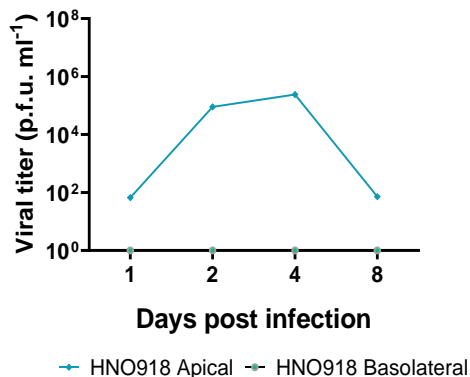**D****RSV/B/BA**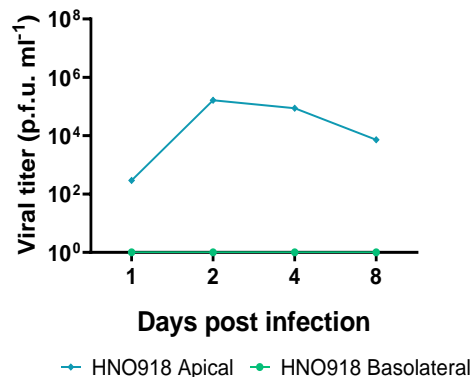

Supplement: FIG S4 [file mbio.03511-21-sf004.pdf]

**H&E**

**PAS/AB**

**Mock**

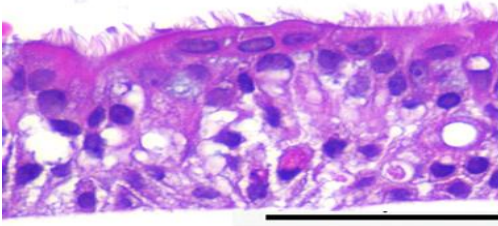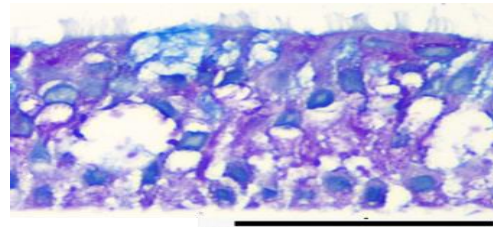

**RSV/A/ON**

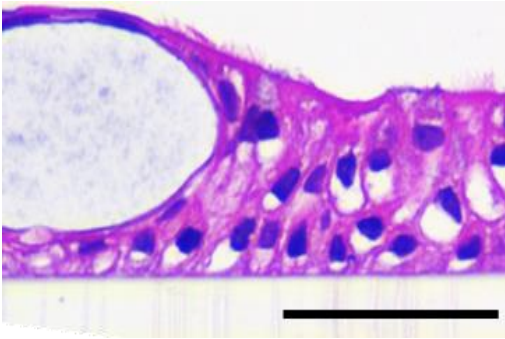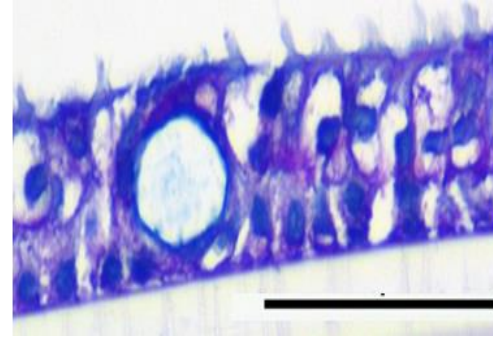

**RSV/B/BA**

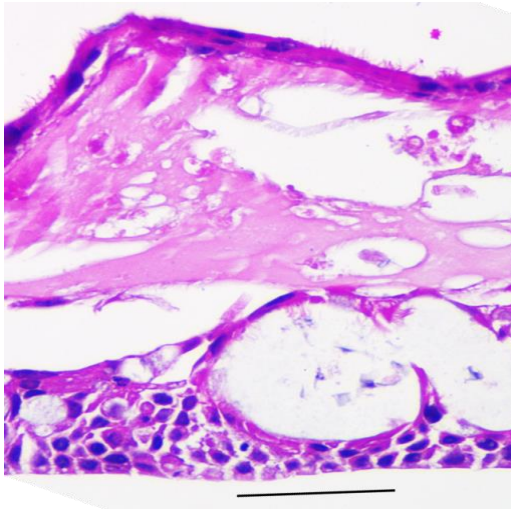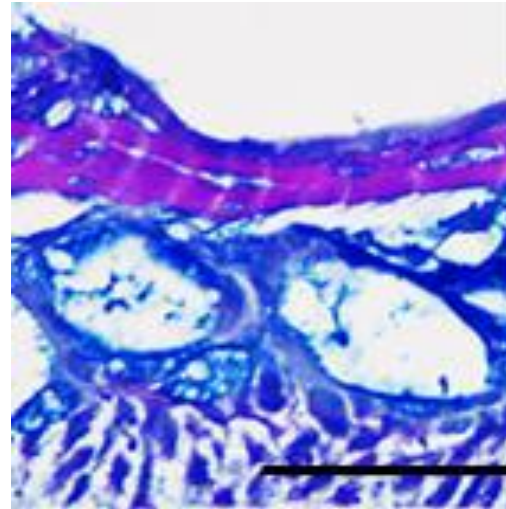

**SARS-CoV-2**

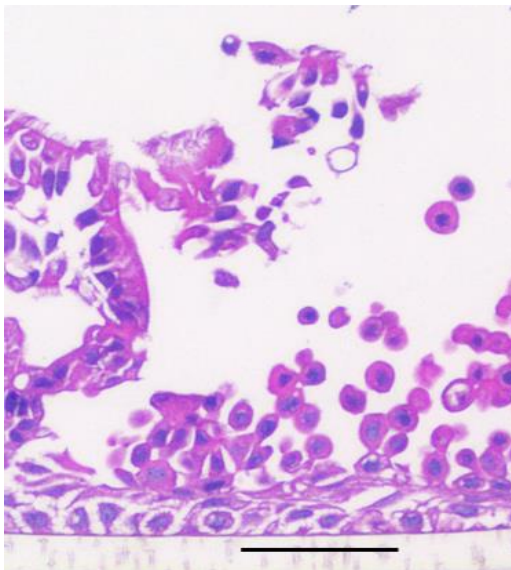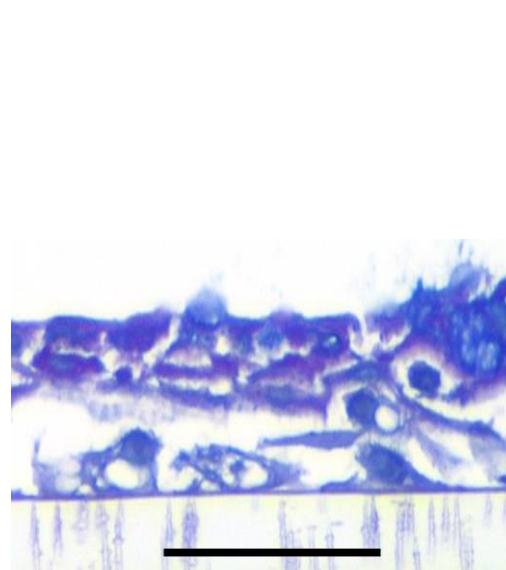

Supplement: FIG S2 [file mbio.03511-21-sf002.pdf]

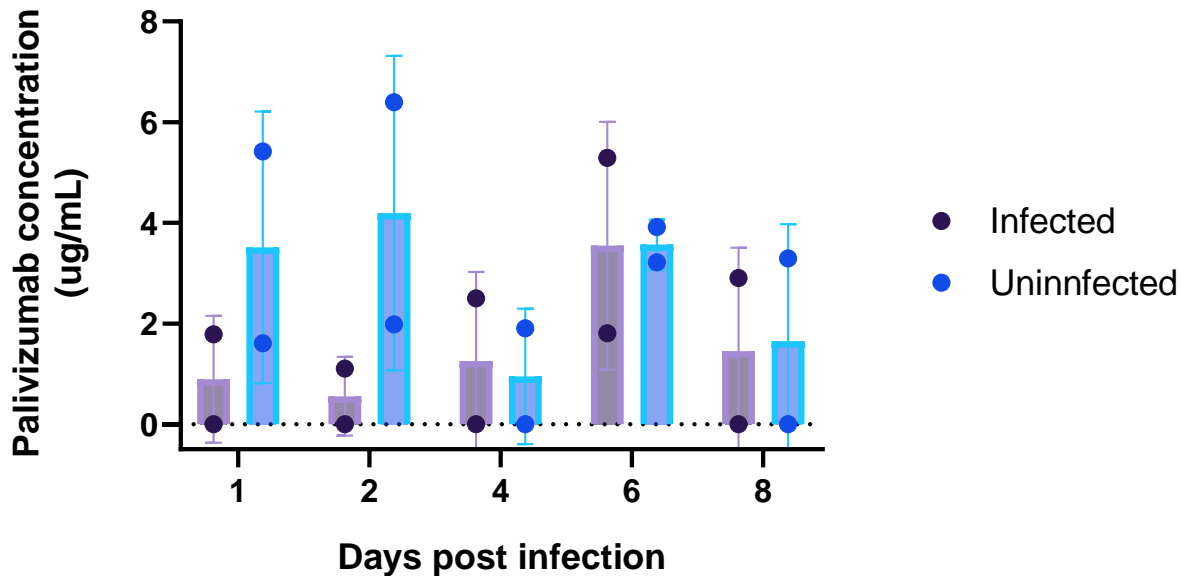

Supplement: FIG S5 [file mbio.03511-21-sf005.pdf]
