# Supplementary material for: The Human Nose Organoid Respiratory Virus Model: an Ex Vivo Human Challenge Model To Study Respiratory Syncytial Virus (RSV) and Severe Acute Respiratory Syndrome Coronavirus 2 (SARS-CoV-2) Pathogenesis and Evaluate Therapeutics
Source: mBio. 2022 Feb 15;13(1):e03511-21. doi: 10.1128/mbio.03511-21 (PMC8844923; doi:10.1128/mbio.03511-21)
Supplement: FIG S3 [file mbio.03511-21-sf003.pdf]

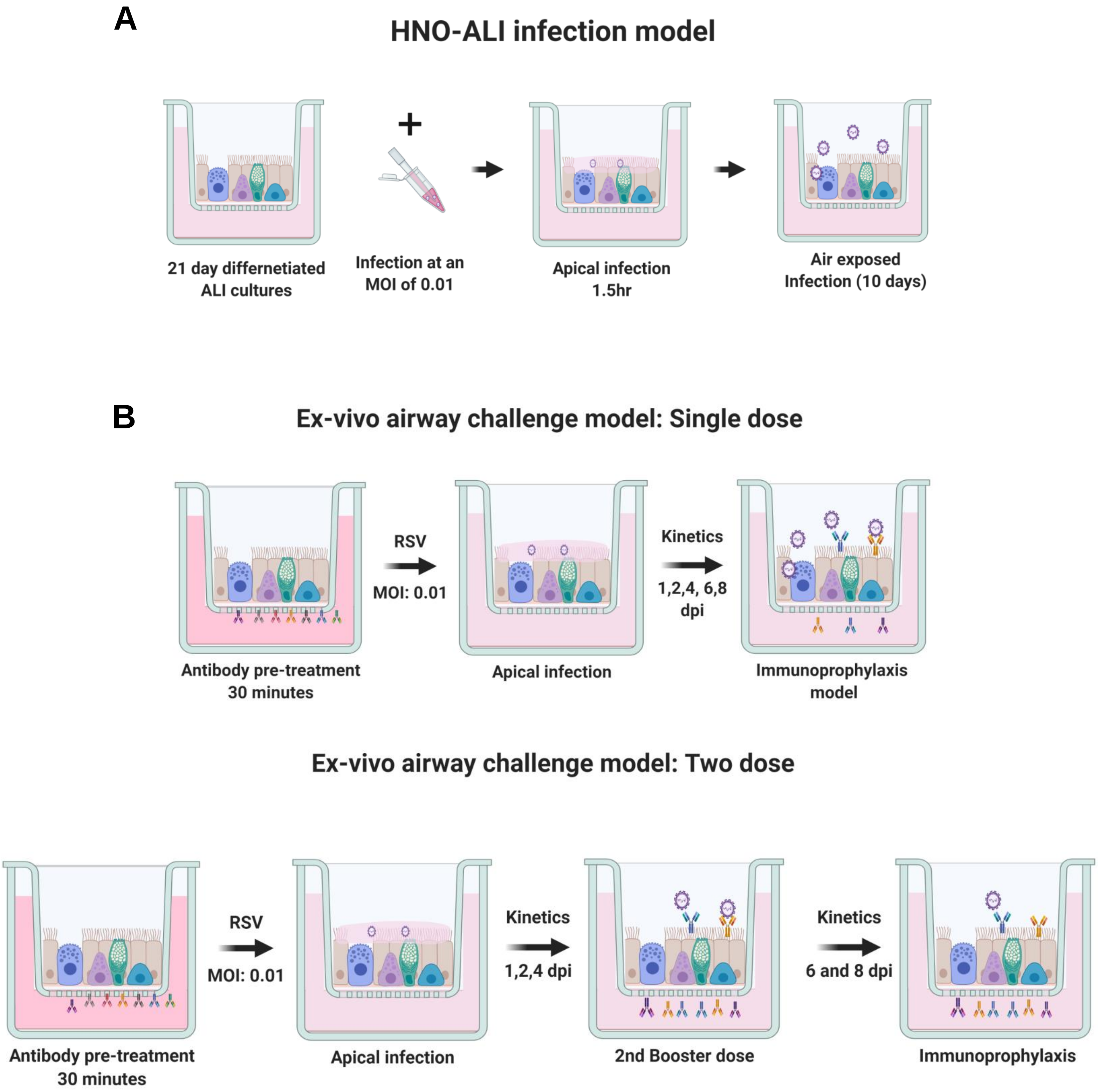
A
HNO-ALI infection model
21 day differnetiated ALI cultures
+
Infection at an MOI of 0.01
Apical infection 1.5hr
Air exposed Infection (10 days)
B
Ex-vivo airway challenge model: Single dose
Antibody pre-treatment 30 minutes
RSV
MOI: 0.01
Apical infection
Kinetics
1,2,4, 6,8 dpi
Immunoprophylaxis model
Ex-vivo airway challenge model: Two dose
Antibody pre-treatment 30 minutes
RSV
MOI: 0.01
Apical infection
Kinetics
1,2,4 dpi
2nd Booster dose
Kinetics
6 and 8 dpi
Immunoprophylaxis
